# Ultra-Wideband Communication and Sensor Fusion Platform for the Purpose of Multi-Perspective Localization

**DOI:** 10.3390/s22186880

**Published:** 2022-09-12

**Authors:** Chunxu Li, Henry Bulman, Toby Whitley, Shaoxiang Li

**Affiliations:** 1 College of Mechanical and Electrical Engineering, Hohai University, Changzhou 213000, China; 2 School of Engineering, Computing and Mathematics, University of Plymouth, Plymouth PL4 8AA, UK; 3 College of Environment and Safety Engineering, Qingdao University of Science and Technology, Qingdao 266042, China

**Keywords:** ultra-wideband, robot localization, sensor fusion, multi-perspective, communication

## Abstract

Localization is a keystone for a robot to work within its environment and with other robots. There have been many methods used to solve this problem. This paper deals with the use of beacon-based localization to answer the research question: Can ultra-wideband technology be used to effectively localize a robot with sensor fusion? This paper has developed an innovative solution for creating a sensor fusion platform that uses ultra-wideband communication as a localization method to allow an environment to be perceived and inspected in three dimensions from multiple perspectives simultaneously. A series of contributions have been presented, supported by an in-depth literature review regarding topics in this field of knowledge. The proposed method was then designed, built, and tested successfully in two different environments exceeding its required tolerances. The result of the testing and the ideas formulated throughout the paper were discussed and future work outlined on how to build upon this work in potential academic papers and projects.

## 1. Introduction

Ultra-wideband (UWB) has been a topic that has existed in one form or another since the turn of the 19th century, starting in 1901 with Guglielmo Marconi using the spark gap radio transmitter to send the first transatlantic radio transmission [1]. As radio communication developed over the 20th century, the concepts that modern UWB utilizes were developed in the form of impulse radar and secure communications to support the US military in the 1960s. In this sense, “it is more appropriate to consider UWB as a new name for a long-existing technology” [2]. In 2002, the Federal Communications Commission in America approved UWB for commercial uses, albeit with bandwidth and power limitations. The authorization sparked other countries to consider UWB and authorize their regional band and power level limitations. 

As research progressed, the use of UWB for applications in real-time location systems (RTLS) became a point of focus, as it could be leveraged to provide a comprehensive and robust solution to improve the localization accuracy of traditional methods, whose primary goal was to build a platform capable of the self-localization of swarm robots [3,4,5]. Those research works also detailed many of the issues surrounding swarm robotics programs, mainly regarding the underdevelopment of onboard positioning and orientation capabilities of said swarm robots relative to one another and their environment. Robot localization is the process of determining where a mobile robot is located with respect to its environment. The authors of [6,7,8,9] demonstrated the capability of several sensor systems which simulated individual swarm robots connected within a local Wi-Fi mesh network. This communication network provided the backbone to allow all the sensor information captured by each system to be combined, using the sensor fusion method to resolve the positions of objects and sensor systems within the area. In detail, the two principal drawbacks were identified as follows: firstly, the system was proposed to use a received signal strength indicator (RSSI) with the Wi-Fi mesh network of the systems. However, this would involve the use of inferred/inference distances, and the test area used was too small for the system to detect a significant signal strength change to calculate range correctly. Secondly, the object detection only worked in two dimensions. Whilst the cameras positioned on the sides of each robot/node could detect the fiduciary ArUco tags on the flat surfaces of the robots for 3D recognition, the mapping/object detection used a 2D LIDAR, limiting the system for the proposed design.

In comparison with other localization technologies, UWB can be classified as an RFID technology. When the area of traditional RFID was reviewed, it was a reasonably affordable system which was not range limited (e.g., ~7 m), whereas UWB systems had the advantage of providing affordable out-of-the-box solutions for an RTLS system [10]. Bluetooth (BLE) comes with meshing and has been successfully tested for RTLS solutions, with the similar issue of there being no readily available commercial products. Moreover, BLE has a lower data rate and lacks the bandwidth scalability to provide an all-in-one solution that is similar to UWB [11]. In the long term, provided that the date rate of UWB could be increased by simultaneously utilizing more of its bandwidth (than just channel 5), its potential could be improved to be on par with Wi-Fi. Laser communications require line of sight and tend to require a large volume of hardware not practical on very small robot applications such as drones. Infrared similarly requires line of sight and is trackable [12].

This paper aims to address both drawbacks by making use of UWB communications rather than Wi-Fi to provide a time of flight (TOF) range measurement that records the distance between transceivers in the system and the mesh network at the same time. This, coupled with a set of 3D sensors (in this case, depth cameras such as those used in the Microsoft Xbox Kinect), theoretically enables improvement upon measuring distance and the insufficient, two-dimensional sensor domain of the original work. This paper aims to assess the ability for these proposed solutions to dispense with the drawbacks noted and can be characterized by the following contributions to tackle the research question.

The interconnection of at least three ultra-wideband modules was developed to enable a communications network in an ad-hoc (drop-in, drop-out) mesh configuration of interlinked nodes. The communications network has been spread over an ultra-wideband frequency spectrum. The network shared the ranging, positioning, and orientation of the system modules connected to the network. The specification demanded that the orientation was measured in increments of at least 1 degree of accuracy. Similarly, relative positioning accuracy fell within 20 cm. The system could communicate with modules at ranges of 10 cm to 20 m. The data rate of the system was determined by the limitations of the UWB communication modules. To supplement the UWB network to facilitate streaming high-bandwidth data such as point clouds, Wi-Fi has been used with a gateway PC for display purposes.

**An object detection and mapping subsystem capable of detecting object(s) or point clouds within at least 5 m range in three dimensions has been developed**. The system could return the location of that object(s) with either spherical, cartesian, or quaternion coordinates. The network of UWB sensors could determine the location of an object, relative to its own position, to within an accuracy of 30 cm or less. The network of sensors captured range data at a rate of at least 2 s per interval.

**A method of combining the above subsystems, controlled through a master computer, was investigated** to provide human user interfaces of the overall system with a series of capabilities, including:

1. Localization of system nodes relative to each other and the local environment.
2. Sensor fusion of all 3D sensors available from each system in the network.
3. 3D world simulation and mapping of the below points:
  - Display of nodes within system range relative to one another.
  - Display distance relative to each node to object(s).
  - Display multilateration of distance information from sensor fusion algorithm.
4. If practical, robot operating system (ROS) deployment for easier development and off-system high-processing-power data analysis.

**A quantifiable environment was designed** to test and verify the effectiveness of the system against its specified requirements. The test area conformed to the following criteria:

- A testing environment that was 5 m in length by 5 m in width by 2.5 m in height.
- Manipulatable objects with measured positions.
- Assumption of no other UWB communication present within the test space.

## 2. Literature Review

### 2.1. Ultra-Wideband Communications and Its Context in Localization

Ultra-wideband has been aptly described by Ghavami et al. as “a new engineering technology” due to the fundamentals that underpin ultra-wideband that have been known and are in regular use by physicists and RF wizards for over a century [13]. Indeed, the real innovation alongside the above-stated governmental legalization is the relentless march of the silicon manufacturers towards cheerfully designing and packing increasingly smaller transistors on chips in vast quantities. This, in turn, has enabled UWB RF systems that can have silicon specifically designed to allow for millions of accurate pulses to be output and received back in a package smaller than a thumbnail [2,13].

The new ease and availability of such systems have allowed researchers and commercial ventures to fully use ultra-wideband (UWB), as previously stated in the introduction. This has several critical advantages over its narrow-band cousins [2,13]. These are as follows:

- The high data rate provided by large bandwidth.
- Ranging data combined with communications data.
- Signal penetration through obstacles using total frequency allocation.
- Low power signals that have superior performance in multipath.
- Low complexity and cost hardware for the RF components.

UWB adheres to Shannon’s channel capacity theorem [14], which states:

(1) $$C=B\log_{2}\left(1+\frac{Signal\;Power}{Noise\;Power}\right)$$

where, as the bandwidth (*B*) of the system linearly increases, so does the data rate or channel capacity (*C*) without increasing the power used by the system. A high bit rate is vital to maintaining real-time systems, with most real-time systems working in the 10 s of milliseconds range. This high speed of communication data through the system can then be leveraged with a large variety of UWB’s TOF ranging techniques.

These can include angle on arrival (AOA), time difference of frrival (TDOA), and two-way ranging (TWR). These use triangulation as their base to break timing, range, signal strength, or angular data down into simple triangle calculations, to compute the positions of each of the units within the system to be known and continuously updating at the real-time rate of the system. This system can be arranged in various forms that functionally represent mesh networks of units, including personal area networks (PANS) and real-time location systems (RTLS), which can move data around and tell where that data is coming from in space. Indeed, there has been IEEE standardization around low-rate wireless personal area networks (LR-WPANs), though not specifically in the field of UWB but as a broad unifier of similar technologies [15].

(2) $$Time\;of\;Flight\;\left(ToF\right)=\frac{T_{round-trip}-T_{reply}}{2}$$

where *T_round-trip_*, also known as round trip time (RTT), is the total amount of time needed for a data packet to travel from its origin to its destination and *T_reply_* denotes the response time of the sensor. In addition to *ToF*, there is another commonly used method to measure the distance between two devices, which is based on received signal strength indication (RSSI). This strategy, however, has a number of drawbacks [16]. The main drawback is that it requires very sensitive equipment to sense the cm level changes in signal strength and COTS hardware will not provide that level of accuracy.

The frequency allocation that UWB resides in has frequencies at the lower end capable of passing through solid objects, such as walls and other obstacles in the environment, due to their long waveforms. This penetration capability and the sheer number of signals output in UWB systems in the region of millions per pulse grants UWB superior performance in multipath environments. Even if a percentage of signals are lost, enough get through to provide a sufficiently accurate impression of the original signal. UWB can operate without interfering with other narrow-band and wide-band services as its imposed power level is so low, which has the added benefit of making it less likely to be intercepted or detected by observers in the areas. The frequency-hopping nature of UWB (carrierless) grants resistance for unintentional jamming.

RF antennas for UWB are simple to design and categorize: “The simplest UWB transmitter could be assumed to be a pulse generator, a timing circuit, and an antenna” [13]. Since this paper was written in 2007, more prominent IC manufacturers have become involved in the market. Despite the increased complexity required to fit the high-speed digital design, the prices have remained relatively constant with ever-increasing capability of newer UWB chipsets. This can be seen in Apple fitting UWB technology into their latest smartphone production models. The company Decawave (now Qorvo) has produced a series of UWB modules specifically designed to set up a two-way ranging real-time location system [17]. As a beacon-based localization system, Decawave modules, acting as anchors, are fixed in place when in use. The first module in the series is the DWM1000, which researchers have implemented to demonstrate the capability of UWB. For example, Kempke et al. describe the construction of a three-antenna system capable of localizing itself within an accuracy of 8 cm median error [18]. Decawave’s next in-series unit, the DWM1001C, provided an out-of-the-box RTLS system built into it. This has captured the attention of several researchers who aimed to categorize the system [19] and show its accuracy and capability. In addition, Jiménez’s work/research is an example of the limitations of both hardware and closed-source software of the DWM1001C (MDEK1001) development board, as well as providing a simple method for improving the capabilities of the system [20].

### 2.2. Simultaneous Localization and Mapping (SLAM)

As the main objective of this paper was to build a system for object detection and mapping, the topic of SLAM became particularly relevant. This is especially evident from the perspective of the ideal shape of this work because what would be produced would be effectively closer to a multi-agent SLAM system using UWB as the localization system. This led to literature that better contextualized the idea of SLAM as a broad overview by characterizing papers that are highly cited or high-level overviews of the topic. For example, Durrant-Whyte and Bailey aptly describe a brief history of SLAM and how different variations on it work in practice [21]. SLAM has been an area of constant research for many years. The field of multi-agent SLAM is the most pertinent to this project, despite the static nature of both the sensor platforms and the UWB system. As the sensor platforms will ultimately make a map of each of the environments, this map must be combined somehow. The concept of map merging in a 3D space is important to the later stages of the project, as shown in this review of multi-agent SLAM algorithms and concepts.

The earliest work found that could be argued as the progenitor of SLAM would be the paper [22] on ‘Shaky’ and the camera system employed to allow it to map objects in its environment. Once mapped, it could then extract ‘ground truth’, or—as stated in the paper—the “floor boundary” for these objects, and avoid them. A particularly innovative SLAM method was the MONO-SLAM paper by Davison et al. [23] which allows the system to need only a single camera to allow it to map key points in its environment. Whilst it may be only tangentially related to this project, reduction in the complexity of required hardware on any robot system is worth looking into as a subject. When in practice, a single swarm robot unit will not be alone and can gain useful consensus on its data from the perspective of all the other units in the system. ORB2-SLAM [24] discusses a partial dream goal idea: creating a SLAM system that is interdependent from the type of sensors it uses. The paper discusses using various types of sensors and still have the SLAM system be fully functional. This would, for example, allow a multi-role mission specification; it is a field that would take the idea of heterogeneous multi-agent platforms to a new level, especially if the resultant data can be unified under a standard layout.

### 2.3. UWB in the Context of Robot Platforms Used for Localizations and Mapping

Significant papers published over several years include: [11,25], culminating in the “Models and Algorithms for use in Ultra-Wideband Localization in Single and Multi-Robot Systems” [26]. This critical work presented methods to use TDOA and a particle filter-based (Monte Carlo) localization method. The discussion surrounding how to implement UWB technology into multi-robot systems involves considerable forethought for how future work in the field would be done and identifies the barriers that need to be removed to fully make use of the technology.

Papers throughout the last decade have incorporated the work that others have done in using Monte Carlo localization (MCL) based approaches to solve completely unknown starting environments with UWB-enabled robots. Examples of this include Prorok, Bahr and Martinoli [25], and Perez-Grau et al. [27]. Indeed, the latter represents a paper that includes some of the key areas of this master’s project, such as the UWB sensors and RGB-D cameras, but rather than use IMU data combined with UWB data, their system used MCL to integrate the point cloud data and the UWB sensor data to estimate robot pose. To test it on more than a single UAV robot would be a viable option for future work related to our paper.

Work has been undertaken to improve what is already there in the sphere of the mathematics supporting robots with Cotera et al. [28] taking the original DWM1000 Decawave modules and using them to improve trilateration algorithms proposed in the 1990s. As time has gone on, researchers have found the relative ease of using commercial-of-the-shelf UWB solutions to provide a platform to quickly implement and test their ideas and algorithms. This can be seen in Hamer and Dandrea [29], where the authors built their system using Decawave modules, then using a Kalman filter to take that UWB data, coordinate the timing of the anchors, and position the drones in space, synchronizing the systems and allowing a group of four to fly in a circle without interacting with one another or knowing each other’s locations. Indeed, Kalman filters are regularly used to fuse sensor data in the form of extended Kalman filters (EKF). Nguyen et al. [30] used COTS UWB technology to successfully fuse sensor date to control four drones flying in a controlled environment. Later, Nguyen et al. [31] took the idea of a UWB as a moveable-anchors space to allow a drone to land on a platform which anchors mark. Combined with onboard camera data, these could be fused to accurately land a drone on a moving platform.

Being able to move anchors rapidly and accurately has been a topic which has captured the minds of researchers, as it gives Decawave modules the ability to be used in more than the static room or building locked scenarios. Almansa et al. [32] proposed the design of a system which would build upon the existing framework of Decawave modules to allow them to be in the above scenario. Their system allows for sub-second and highly accurate recalibration of anchor positions after being moved into a new location. This has fascinating implications for future work for this project, surpassing the limiting factor of fixed beacon-based localization. An earlier paper by the same group [33] created a data set for the community of researchers to use in their experimentations.

The impression illustrated by the literature is to create an ad-hoc, decentralized auto-calibration subsystem for swarm robot platforms that can be added to any type or shape of platform, making its application heterogenous in the form of a mesh network. The ability to grow and shrink the number of units seamlessly within the system—ideally without upper limits—may be a goal with wide-reaching impacts and implications. Indeed, the above work shows a gap in both the research and the market for a generic system with built-in methods for adding in unifying modal sensor data.

## 3. Methodologies

### 3.1. Design

The authors had already considered the shape of the hardware during the proposal, defined as three main sensor platforms at the time of completion. Each platform consisted of three principal hardware components: UWB communications module, 3D imaging sensor, and microcomputer. These were supported by ancillary components: cabling, battery, 3D-printed case, and tripod, and finally by human interface devices: keyboard, mouse, and display. For the stretch goal, a UWB radar module would also be required. Each of the main hardware components will now be addressed.

#### 3.1.1. UWB Communications Module

The Decawave (now Qorvo, Greensboro, NC, USA) DWM1001C Development Board was selected on the initial bill of materials, due to the regular reoccurrence of the DWM1000 UWB transceiver in literature and its relative low cost. It provides a system centered on the DWM1000 ultra-wideband (UWB) transceiver IC, controlled by a Nordic Semiconductor nRF52832 system-on-chip providing both microcontroller and Bluetooth (BLE) interface. Ancillary is a 3-axis accelerometer STMLIS2DH12TR and DC–DC converter for conversion from input 3.3 V to 1.8 V, as described by the below diagram from the user documentation.

**Figure 1:**
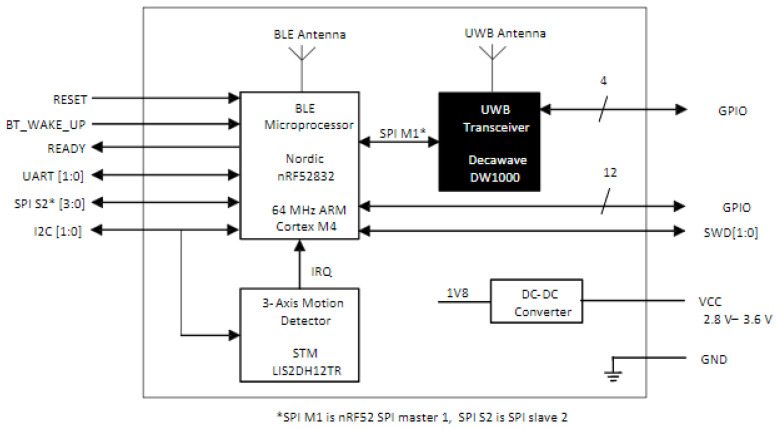
Block diagram of DWM1001 module [35].

**Figure 2:**
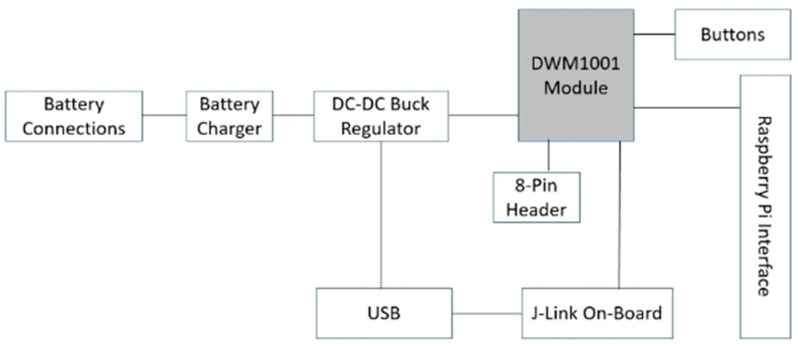
Block diagram of DWM1001-DEV/MDEK1001 module [35].

**Figure 3:**
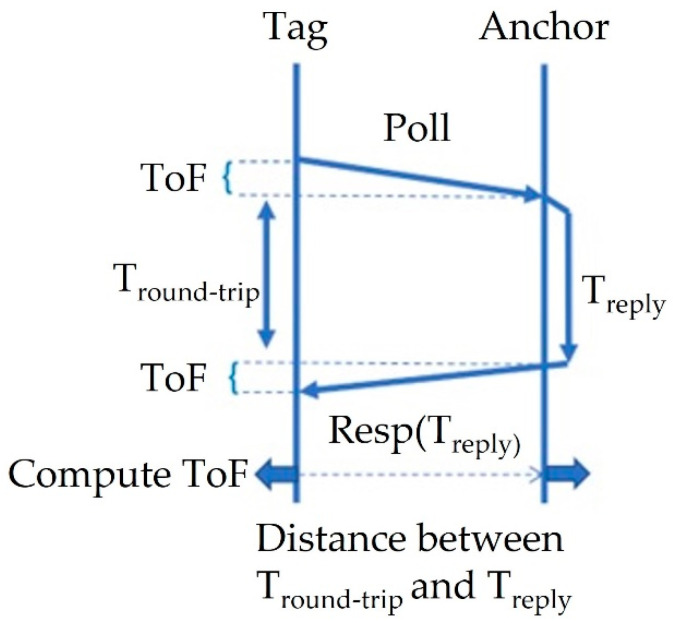
Time of Flight Diagram.

The development boards provide interfacing/programming options for the units and power/battery management for all systems. The DWM1001C modules can be flashed via the dev board J-Link and controlled using SPI, UART, I2C, and Bluetooth, via the micro-USB port or the Raspberry Pi 26-way pin header, as can be seen in the below diagram (Figure 1 and Figure 2) [34]. The DWM1001 module runs a two-way ranging (TWR) real-time location system (RTLS) on its firmware. The system allows the module to be configured as an anchor, a rag, or a gateway within the RTLS. The anchors act as fixed nodes within the system, providing a reference point for the system to localize. The tags are the mobile portion of the system that use a single-sided two-way ranging scheme (see Figure 3). This lets the tag range between 3 or 4 anchors, and then calculates its position relative to those anchors. 

**Figure 4:**
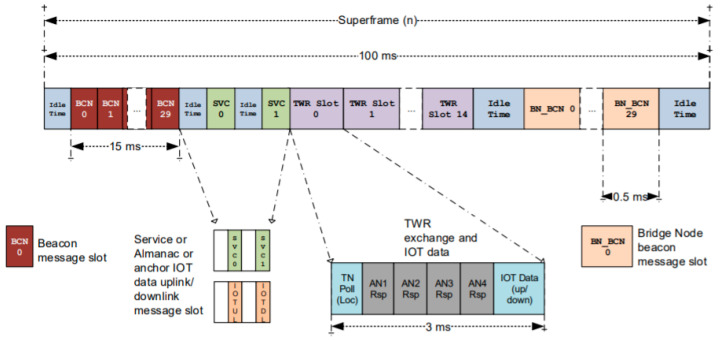
Decawave RTLS—Superframe Structure [36].

The units use the channel access method time-division multiple access (TDMA) to slot each of the TWR transmissions between anchors and tags into their position within a TDMA superframe (Figure 4), which is essentially a method of synchronizing each node in the system to let each of them transmit during their assigned slots within the superframe. Techniques for avoidance, detection, and resolution of collision of asynchronous messages allows the DWM1001 firmware to support an extensive array of modules up to 15 tags at the full update rate of 10 Hz each in a 150 Hz frame, which is twice as many as the number required to support this project. The full system can support 30 anchors and 9000 tags, albeit at a drastically reduced update rate [36].

#### 3.1.2. Imaging Sensor

The Intel RealSense D455 is a stereo vision depth camera system comprising two imaging sensors, an infrared pattern projector, an RGB camera, and an IMU. The vision components are then unified by the Vision Processor D4, which can combine all the data into a color depth image, entirely controlled through USB 2.0/3.1. The reason for selecting an RGB-depth camera over a regular RGB camera is straightforward. SLAM and multi-agent SLAM can be done with regular web cameras, but the issue is how that information is inferred and translated into a form manipulatable in a 3D model. As sensor fusion platforms, the depth camera allows exact distance readings on each pixel detected by their sensors. Most COTS systems output a point cloud, which has a set of points with coordinates located in the space of the system localized to a camera frame or a world frame.

Stereoscopic vision in depth cameras such as D455 use the principle of triangulation in the form of epipolar geometry to reduce a complex two-dimensional problem into a one-dimensional one. It does this by seeking matching key points or features on the two cameras, which are paired as intersection points and estimates the disparities between them, allowing depth to be calculated. The stereo view of the world must be aligned so each camera’s data is in parallel to one another. Most stereo depth cameras are designed to be parallel at the expense of specific lens manipulations, but this eliminates the need for post-processing to create parallel views. This point detection is crucial at speeding the searching up as it reduces the number of parameters that the system needs to store to calculate where the cameras are with respect to an object and possibly each other. In the visible spectrum of light, key points or features are seen the same way humans recognize corners, textures, or edges. These features can also be added by artificial pattern generators such as IR projectors.

There are three cameras on the D455: two identical “imager” cameras that can detect both infrared and visible light and display that information in monochrome and a “color sensor”, a standard RGB camera. In the case of the D455, the two images are wide angled with large overlapping fields of view. The RGB camera can produce synchronized color images that can be overlaid over the point clouds produced by the system to create RGB point clouds, with each pixel hue value filled in. The onboard infrared projector allows the camera to apply an infrared pattern to surfaces that do not have distinguishing features, for example, a large white wall. By projecting an infrared pattern on the wall, the depth cameras will now have a known texture (in the form of equally spaced points of intense infrared light which appear white in monochrome) to perform its depth calculations. This has the added advantage of being able to use the camera in low-light situations. The depth field of view at distance (*Z*) at any distance (*Z*) can be calculated using the equation below:

(3) $$Depth\;FOV=\frac{HFOV}{2}+\tan^{-1}\{\tan\left(\frac{HFOV}{2}\right)-B/Z\}\;$$

where *Depth FOV* denotes depth field of view, *HFOV* represents horizontal field of view of left imager on depth module, *B* is baseline, and *Z* is distance of scene from depth module.

#### 3.1.3. Single-Board Computer

The above two main components/subsystems would need to be linked to allow both to be run in unison and unify their results locally within the same robot platform. This fusion of data could then be sent over the network for other uses. Optimally, running the UWB module and the RGB-D camera on laptops with suitably modern hardware would maximize the system’s overall performance. However, this was nixed favoring single-board microcomputers reducing the cost to ~100 pounds per unit. The two single-board computers with well-documented backgrounds using the robot operating system (ROS) were the Nvidia Jetson Nano 4 GB and the Raspberry Pi Model 4 8 GB ram.

A Jetson and Raspberry Pi were compared against one another by their performance of running a D455 with ROS and the 3D visualization environment—rviz. The Jetson Nano performed at 15 frames per second (FPS) compared to the Raspberry Pi at 5 FPS, leaving the Jetson Nano as the single-board computer in this paper. The Nvidia Jetson Nano is a 260-pin SO-DIMM (NVIDIA, Santa Clara, CA, USA, 2019) (small outline dual in-line memory module) module that is built around the Nvidia Tegra X1 series system on chip (SOC); both versions (A02 and B01) of the development carrier board that holds the module were purchased for this project. In combination with carrier board and module, the Jetson Nano, referred to as a whole from here on, is a powerful single-board computer designed to provide AI and IoT embedded applications capable of running a 64-bit operating system. The Jetson Nano comes with a Linux Image specifically designed to run on the platform. It is not surprising that Jetson output performed the Raspberry Pi specifically geared to provide a high-performance integrated graphics processing platform (using 128 CUDA cores) using only 10 watts of power.

#### 3.1.4. ROS Melodic

Due to the requirement to use Nvidia Jetsons, which provide an image that uses Linux in the flavor of Ubuntu 18.04 LTS (Bionic Beaver), this work has used the twelfth robot operating system (ROS) distribution Melodic Morenia. In the academic world, ROS is becoming a de facto standard for robot software. The practicality of sharing, ease of integration, and implementing cutting edge research from other researchers in the world has been leveraged successfully in this paper, thus allowing the use of algorithms and hardware that would have taken teams of people years to write and test. Each of the ROS software packages used in this paper are as follows:

- **RealSense SDK 2.0** provides the backbone for running all the different types of cameras in the RealSense series to provide a variety of functionality to support most projects using either depth or tracking cameras made by Intel.
- **DWM1001 ROS Package** provides a python interface with DWM1001-DEV board to the ROS transform “tf” package 3D coordinate frames, allowing the positions of the Decawave modules with RTLS tied to (0, 0, 0).
- **The Point Cloud Library** is an open-source, standalone library designed for the processing and manipulation of 2D/3D images and point clouds.
- **Octomap Package** is fully ROS integrated and provides an efficient and simple way to implement a 3D occupancy grid mapping system, allowing its setup entirely from a launch file.
- **ROS TF Transform** allows ROS to display multiple coordinate frames with respect to time.
- **April Tag 3** is a comprehensive open-source visual fiducial marker detection algorithm [37]. The function of the tags is to provide a precise 3D position that can be referenced against the point cloud. This ROS wrapper drew this project’s attention due to its easy implementation, for example, the markers edge length can be easily customized in the set-up software to suit any system. Early testing of the package showed that the system could easily detect 12 tags close to one another up to a range of 2 m, with the tags being 4.15 cm to an edge. These are characterized in ROS as 3D coordinate frames representing each detected tag from the perspective of the camera topic provided to the package.
- **IMU Tools**: Madgewick Filter transforms the acceleration (m/s^2^) and angular velocity (rad/s) into orientation quaternion data in the ROS sensor_msgs/Imu.msg field; Rqt_reconfigure program was built to create a measurement tool and offset fixer that allowed an “alignment frame” to be generated.

### 3.2. Unification

Once the above hardware and packages were integrated and tested on either the Nvidia Jetson or the laptop acting as the ROS master and processing hub, these were then unified with the below plan on feeding data from the sensor platforms to the laptop.

#### 3.2.1. Sensor Platform

**Figure 5:**
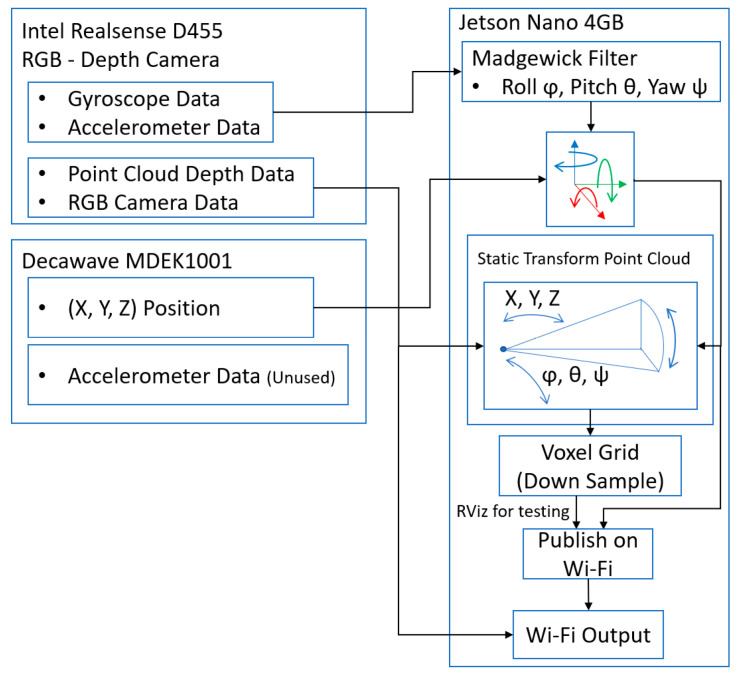
Base system—Jetson Block Diagram.

The Jetson Nano is connected to MDEK1001 and the D455 RealSense Camera via a USB to Micro-B USB and USB 3.0 to USB-C, respectively. All sensor platforms are pointed in the direction of the world frame; they are then rotated towards the test area once all their packages are turned on. These are connected as follows, shown in Figure 5:

The gyroscope and accelerometer data in the form of angular velocity and acceleration are passed to the Madgewick filter to output a 3D coordinate frame called imu_alignment_frame. This can then use a static transform publisher to position the point cloud frame “depth_colour_frame” on top of it in the corrected orientation relative to the camera link (the camera group hub of D455 frames). This produces a movable- in-orientation point cloud view of the area currently in front of the camera relative to the camera link. At the same time, the Decawave module has its position read by the package and the world frame at (0, 0, 0) coordinate created with real anchors and the tag module positioned relative to the world frame. Using another static transform, the camera link is positioned onto the model position of the tag. This allows the point cloud to move in sync with the tag as it is updated. The point cloud output by the D455 is bandwidth-intensive, so it is down sampled using a voxel grid, with a leaf size set between 0.01 and 0.05. At 0.03, the bandwidth requirement calculated by ROS is reduced from 100 MB/s to 10 MB/s. This down sampled point cloud, which is moveable in orientation and position, is then sent over the Wi-Fi or is tested on the Jetson within rviz.

#### 3.2.2. Laptop

**Figure 6:**
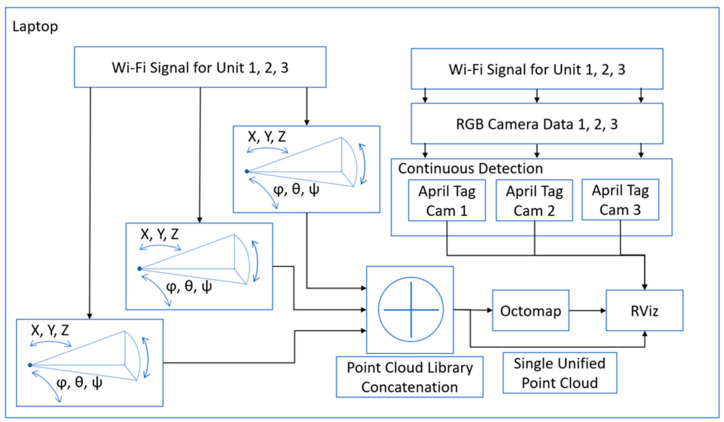
Base System—Laptop Block Diagram.

The moveable point clouds from the three Jetsons in the system are received over the Wi-Fi on the laptop. The point clouds topics are passed to the PCL concatenation algorithms, which turns them into a single point cloud. This point cloud can then be passed to Octomap to begin building a map of the environment as the cameras move around in orientation and position and the results are displayed in rviz. Rqt_reconfigure is used to manually align the point clouds together with respect to camera one. At the same time, the compressed camera image topics from each camera are passed to 3 duplicate April tag packages. This begins a search for tag 0–3 in their images. If detected, the transforms of the April tags are then placed into the system at the correct position in space relative to the camera that saw it; this should be superimposed upon the combined point cloud. A diagram of this is shown in Figure 6.

Hector trajectory mapping can be used to map the trajectory from their starting position in the environment. If the aligned_depth_to_colour_frame is fed to the hector trajectory mapping, the system will also include the orientation at each measured stage of the trajectory line, not just positions if the tag is used. A copy of the full rqt_graph has been enclosed as a supplementary material of the paper (Figure S1).

#### 3.2.3. Subsidiary Design

**Figure 7:**
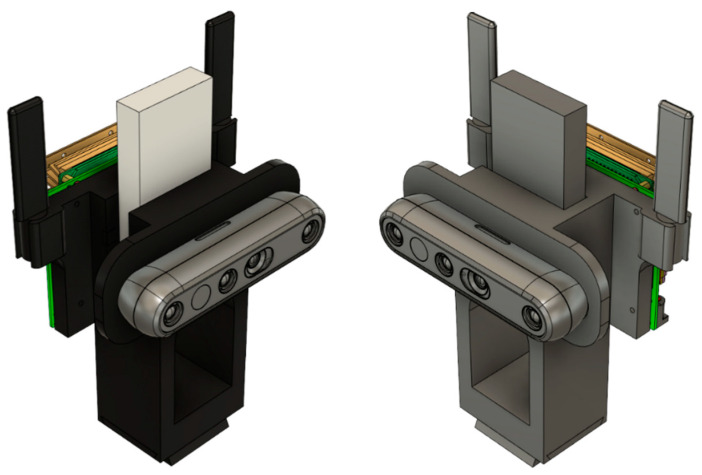
3D Models of Case with Hardware.

A 3D-printed housing (see Figure 7) was designed using the simple expedient of downloading each 3D CAD model of the system’s three main components and importing them in Fusion 360. Once imported, the fixing locations of each of the components were found, and then a flat plate with the relevant hole locations extruded was aligned with the D455 camera at the front, the DWM1001-DEV in the middle, and the Jetson at the back. This design was tested out, and it was considered more practical to use the MDEK1001 chassis slotted into the side of the solid model, with two attachment points for the camera on the front and the Jetson on the back. After dimensioning the MDEK1001, the final design, as seen below, was implemented with a tripod stand dovetail attachment point offset at the maximum length of the display port jack attached to the Jetson plus four centimeters. This large block was then holed to reduce printing material amounts.

## 4. Experimental Studies

To satisfy the fourth contribution of the paper, the system would need to be categorized within a 5 × 5 × 2.5 m test space. This test space would have manipulatable objects which could be used to test the capabilities of the system. All 3D coordinate frames were positioned relative to the world frame. This has the advantage of tying in both orientation and positions of all data as individual frames with a global reference point, without displaying segments of the data not relevant by themselves (e.g., gyroscope output). This data unification also allowed the use of the tf suite of commands, such as the ability to query for specific transforms between frames and track the frames through time inside rviz.

Tied to some of the frames were point clouds generated by the depth cameras. Point clouds represent a matrix of coordinate data points in space (in this case, 3D space), which are also tied to the world frame via their origin frame. The majority of the data used in the system was generated from the point clouds.

### 4.1. Set-Up

**Figure 8:**
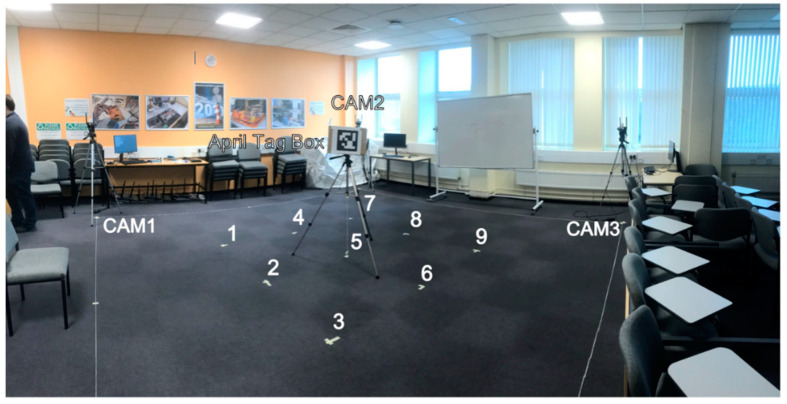
Annotated Test Area.

To set up the test area for the project, two spaces would have to be set up, one over the other, to support the project. The first would be the area described in contribution four as a 5 m × 5 m × 2.5 m test space, and the second, the fixed anchor locations of the RTLS network. The test area was made by taking a tape measure and measuring a square 5 m to a side. This was then marked out using a string cut to length, and the center of the square was specified at the (2.5, 2.5) coordinate. Around this, in a dotted square pattern, was a measured set of marker points, 1 m away from the center point. The space was then verified again using a laser tape measure. This was to establish an error-free ground truth for the system as the best possible physical measure of the space, given the current constraints of human error (See Figure 8).

The anchors were set up using a laser tape measure to measure the height (*z*-axis) of the anchors from floor to ceiling with a suitable offset to account for the center of the MDEK1001 chassis. This value was shown as 2.85 m. (*x*-axis, *y*-axis) coordinates of anchors measured using a laser ruler are as follows:

- Initiator Anchor “Orange” at (0, 0) coordinate.
- Anchor “Pink” (7.13, 0) coordinate.
- Anchor “Blue” (0, 7.05) coordinate.
- Anchor “Yellow” (7.128, 7.15) coordinate—this is due to the room not being completely uniform regarding optimal positions for the anchors.

**Figure 9:**
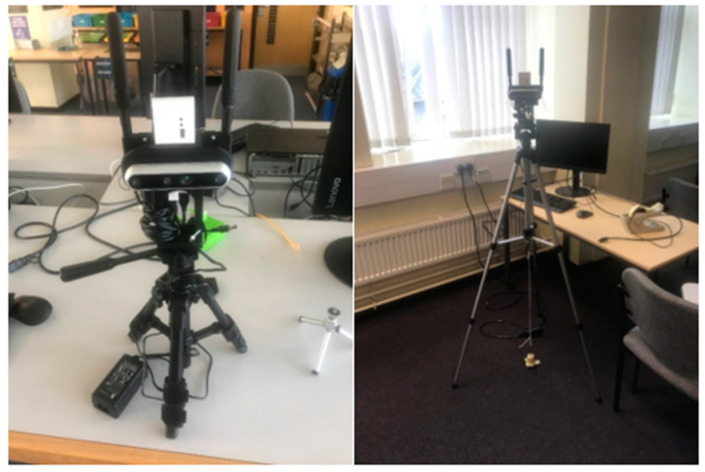
Sensor platform (fully built) and camera 3 with weight alignment.

The camera units of the system were located by placing their tripods over the corners of the test area. The camera units center point was positioned relative to the corner, using a weight on a string on the tripod (see Figure 9). The cameras were levelled using a two-stage method of aligning the two spirit levels built into the tripods for leg length and pitch verified with a digital laser level (RockSeed LV1).

### 4.2. Method for Measuring

As the measurement area and its baseline ground truth were established, the system’s performance could now be categorized to test the alignment accuracy of the cameras relative to their point clouds and measured. To do this, there was a two-stage measurement process. The cameras were set up as detailed in the technical description, and the April tag package was set up to detect tags 0–3. These tags were then attached to a cardboard box with 20 by 20 by 30 cm dimensions. This allowed the cameras to report the tag positions that they detected relative to their camera reference frame. The cameras pointed toward the center of the test space from their positions at the corners. The April tag box was placed on each of the nine marked locations at the center of the test area, labelled 1 through 9 left to right from the start at the bottom left-hand corner (See Figure 9).

As the April tag box was moved between the nine positions, the tags detected were recorded as relative positions to the cameras that detected them. For simplicity, it was ensured that tag 0 was measured by camera 1, tag 1 by camera 2, and tag 2 by camera 3. A laser ruler (DTAPE DT50) was aimed, from each camera sensor, at the center of the April Tags, where transforms are created in the program and the value measured. The results of the relative position of tags 0–3 to cameras 1–3, at positions 1–9, were recorded along with the laser tape measure data at positions 1–9. This concludes the qualitative testing method.

### 4.3. Experimental Results

What follows are the results of the test detailed in the previous section. The system results are simple to interpret: the laser ruler’s data is effectively the hypotenuse of a right-angle triangle from the camera position to the April tag box in 3D, so this data does not need to be modified before use. The relative position data for the camera to the tag must be run through a 3D Pythagorean theorem equation before it will return a *3D Hypotenuse* that can then be compared to the laser ruler data. The formula for this is as follows:

(4) $$3D\;Hypotenuse=\sqrt{\left(x^{2}+y^{2}+z^{2}\right)}\;$$

where (*x*, *y*, *z)* denotes the coordinate of a point in Cartesian space.

**Figure 10:**
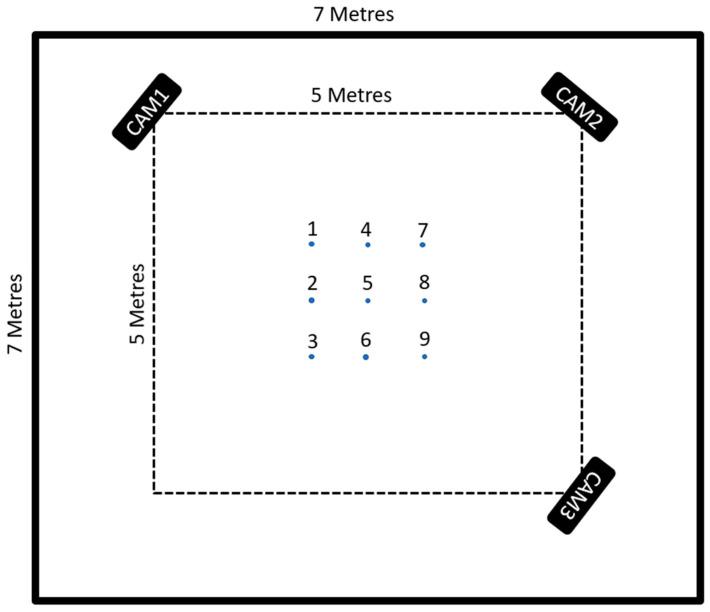
Layout of space.

The data was recorded into Excel for faster manipulation and display. Table 1 shows the values, and Figure 10 illustrates their distribution under three cameras. The results were compared to positions 1 through 9, and their values were displayed as scatter plots.

**Figure 11:**
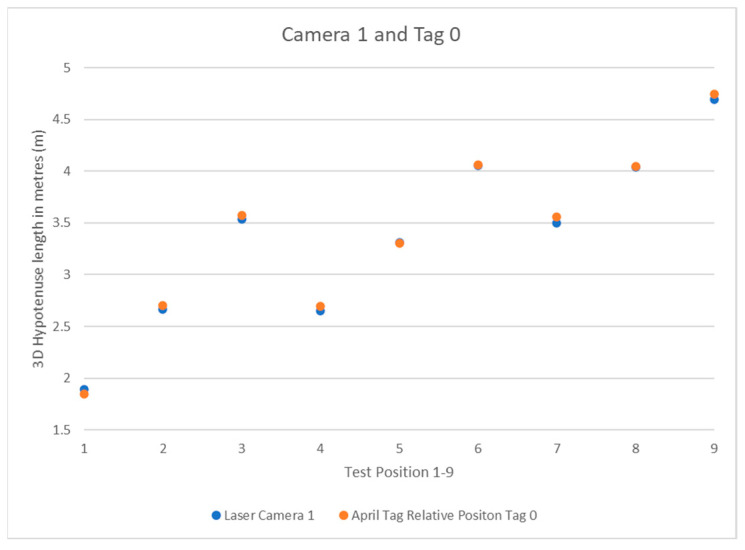
Camera 1 and Tag 0 Position Results.

**Figure 12:**
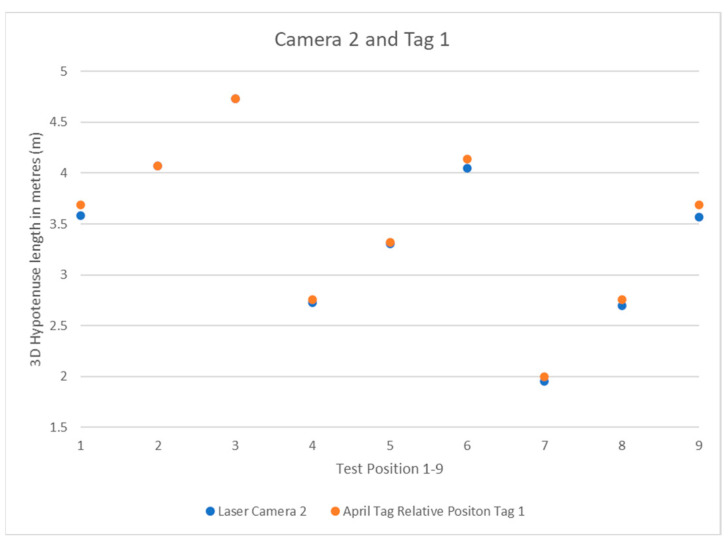
Camera 2 and Tag 1 Position Results.

**Figure 13:**
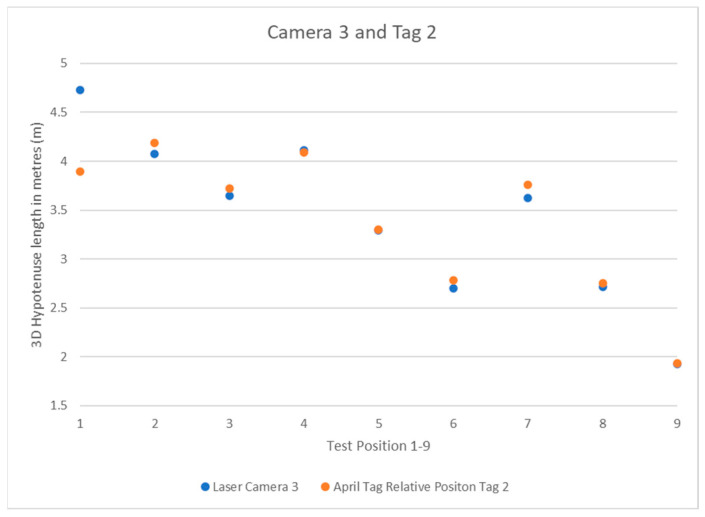
Camera 3 and Tag 1 Position Results.

The results show the system’s expected pattern from each camera’s perspective, from the known quantity of the positions in the square-dotted pattern used to place the tripod. Each camera has a pattern that can be interpreted as to its perspective. As the system is left to right going upwards in the 1–9 April tag box positions, camera 1 in Figure 11 can be closest to the April tag box and moving away as the position number increases. Camera 2 in Figure 12 shows the same 3-stage pattern but split from the camera’s perspective on the “middle” corner between cameras 1 and 3 from the clockwise perspective beginning at camera 1. Camera 3 is mirrored on camera 1, with it starting further away and getting closer, as seen in Figure 13.

The previous graphs were in meters and the actual error calculated for the system was too small to accurately show on such a large range. The above box-and-whisker plot was made to show the difference error between the laser tape measure and the relative tag position reading from the perspective of each camera. Camera 1 represents the most stable data with an upper and lower quartile difference of 5 cm, with the maximum and minimum just over 10 cm apart. The camera has its data values closest to zero but slightly weighted on the negative side. Cameras 2 and 3 have similar sizes of boxes, with a 10 cm difference in their upper and lower quartile with larger errors between the laser data and the relative tag position data. The major difference between camera 2 and camera 3 is the large 83 cm outlier. This has been removed from camera 3 as it distorts the entire diagram.

**Figure 14:**
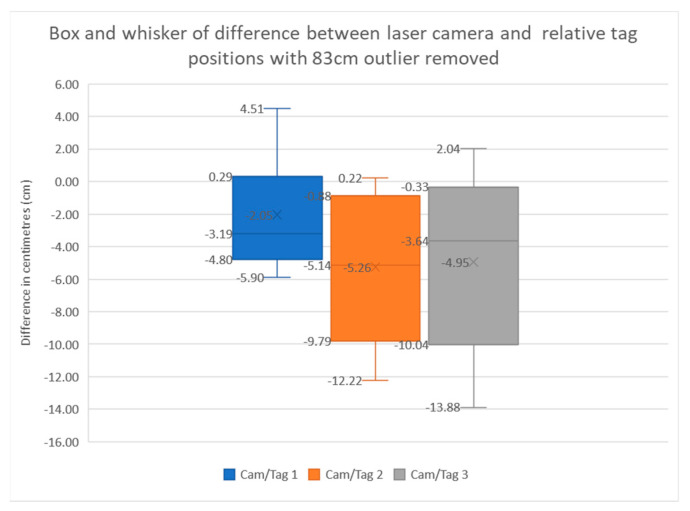
Box−and−whisker plot described by its title.

The percent error has been calculated by camera, with camera 1 being 1.1%, camera 2 being 1.67%, and camera 3 either being 1.6% without its outlier or 3.57% with it included (shown in Figure 14). After all the errors have been normalized, the average overall error is 4.69 cm, excluding the 83 cm outlier, and 7.79 cm with it included. There has been extensive testing/debugging of the system throughout building and testing, albeit without a specific test method in place. It has thereby been visually verified that elements of the system are working within the specification defined by the ROS packages that run them. The relevant visual results can be found from the enclosed videos.

## 5. Conclusions

This paper answered the research question—can ultra-wideband technology be used to effectively localize a robot with sensor fusion?—by making four contributions. The main aim has been to build a set of networked sensor platforms, which has since been developed, tested, and verified in a quantifiable environment. The system is a unification of 3D sensor data and IMU data from the D455 RealSense RGB-D camera. The UWB localization system provided by the MDEK1001s in their RTLS arrangement, and the Jetson Nano providing the electronic ‘glue’, unifies both modules and connects them to the Wi-Fi network with other platforms in the system. Contribution 1 was based around using an ultra-wideband drop-in/drop-out RTLS network backed up with Wi-Fi for large data transmission, and the accuracy throughout remains <5 cm. Contribution 2 was to develop a 3D sensor subsystem capable of mapping and object detection, and it was found to be capable of working accurately at the largest length present in the test area of 7.5 m with the maximum error at 13.88 cm, which is less than half the minimum required value of 30 cm. Contribution 3, to combine the systems and control them through a master computer, has been accomplished using the ROS as the base control platform. For contribution 4, the April tag box, chairs, whiteboards, and people provided moveable objects within the environment for the system to detect and map with the average error being half as much as the required accuracy of the system.

This technology can be used in any indoor robot localization system, such as for large warehouses or maid robots (cleaners, vacuums, hotel staff); the systems are practical in outdoor environments provided the inverse system is applied. For example, the robots carry the anchors (with appropriate mathematical changes to the range finding) and the humans/other robots have the tags. The large bandwidth, if synced correctly, allows multi overlapping meshes of anchors and tags as the robots connect to one another within their environment. In the short term, this can be represented as simply as a large outdoor robot such as a tractor being able to follow and avoid a human with a tag.

However, the MDEK1001 has a range of 200 m (in best conditions); this is a power-restricted range and, for general use, is unreliable, which restricts the total operational range to 20–30 m reliably. Interestingly, this technical limitation of the low-power units is entirely a design choice, which is defined by the intended use of the hardware. Subsequently, if researchers take the time to develop a UWB system that has a variable power level and a full bandwidth use of the technology (rather than being limited to channel 5–500 MHz) but a multi-GHz bandwidth, the system would get a huge data rate and range increase in a single stroke [38]. Although, admittedly, this is well beyond the scope of this paper, it is possible in theory; the modern application of UWB that is currently in industry use, i.e., robot positioning and use in smart phones, simply did not require this range so the hardware did not develop down this path. Inspired from the literature [39], the future direction should be to develop a combined UWB communications module and UWB radar module to create a single solution using only UWB technology.

## Figures and Tables

**Table 1:** Testing Data Display.

	*x*	*y*
Position 1	1.5	1.5
Position 2	2.5	1.5
Position 3	3.5	1.5
Position 4	1.5	2.5
Position 5	2.5	2.5
Position 6	3.5	2.5
Position 7	1.5	3.5
Position 8	2.5	3.5
Position 9	3.5	3.5

## Supplementary Materials

The following supporting information can be downloaded at: https://www.mdpi.com/article/10.3390/s22186880/s1, Figure S1: Full rqt_graph output of base system.

## Statement

Chunxu Li and Henry Bulman are co-first authors. The paper was written based on the MSc dissertation of Henry, who has graduated with distinction and is currently a senior robot engineer at Babcock International, UK, and Chunxu was responsible for the manuscript’s drafting, editing, revisions, and organizations. Moreover, Chunxu was the supervisor and funding supplier for this work.
