# Supplementary figures and images for: Ultra-Wideband Communication and Sensor Fusion Platform for the Purpose of Multi-Perspective Localization
Source: Sensors (Basel). 2022 Sep 12;22(18):6880. doi: 10.3390/s22186880 (PMC9504988; doi:10.3390/s22186880)

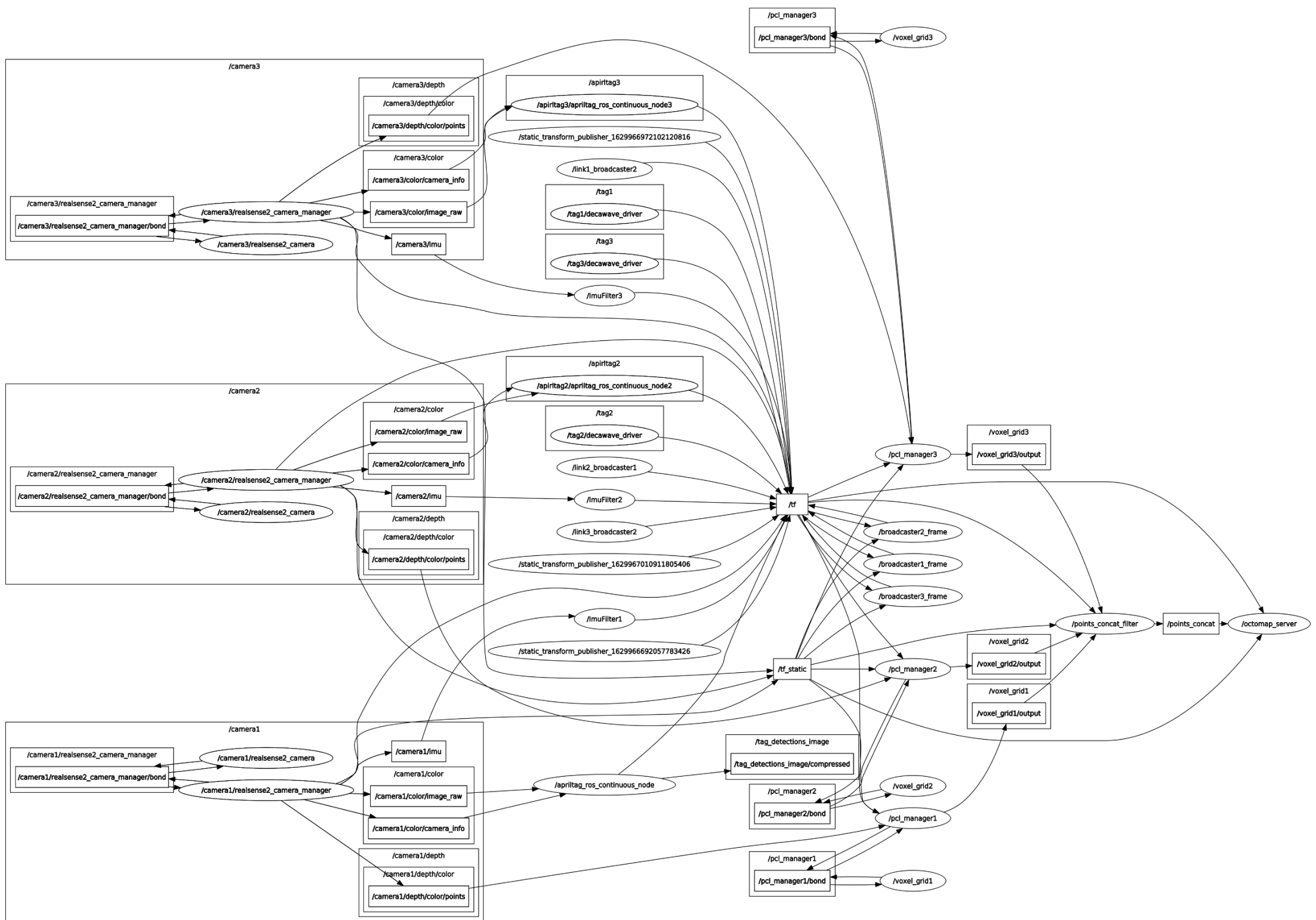

Figure S1. Full rqt\_graph output of base system

Supplement: Supplementary file 1 [file sensors-22-06880-s001.zip › sensors-1821589-supplementary.pdf]
